# CORM-A1 Alleviates Pro-Atherogenic Manifestations via miR-34a-5p Downregulation and an Improved Mitochondrial Function

**DOI:** 10.3390/antiox12050997

**Published:** 2023-04-25

**Authors:** Hitarthi S. Vyas, Ravirajsinh N. Jadeja, Aliasgar Vohra, Kapil K. Upadhyay, Menaka C. Thounaojam, Manuela Bartoli, Ranjitsinh V. Devkar

**Affiliations:** 1 Chronobiology and Metabolic Endocrinology Lab, Department of Zoology, Faculty of Science, The Maharaja Sayajirao University of Baroda, Vadodara 390002, India; vyashitu25@gmail.com (H.S.V.);; 2 Department of Biochemistry and Molecular Biology, Augusta University, Augusta, GA 30912, USA; 3 Department of Internal Medicine, Division of Gastroenterology and Hepatology, University of Michigan, Ann Arbor, MI 48104, USA; 4 Department of Ophthalmology, Medical College of Georgia, Augusta University, Augusta, GA 30912, USA

**Keywords:** miR-34a-5p, atherosclerosis, CORM-A1, mitochondrial respiration, oxidative stress

## Abstract

Atherogenesis involves multiple cell types undergoing robust metabolic processes resulting in mitochondrial dysfunction, elevated reactive oxygen species (ROS), and consequent oxidative stress. Carbon monoxide (CO) has been recently explored for its anti-atherogenic potency; however, the effects of CO on ROS generation and mitochondrial dysfunction in atherosclerosis remain unexplored. Herein, we describe the anti-atherogenic efficacy of CORM-A1, a CO donor, in in vitro (ox-LDL-treated HUVEC and MDMs) and in vivo (atherogenic diet-fed SD rats) experimental models. In agreement with previous data, we observed elevated miR-34a-5p levels in all our atherogenic model systems. Administration of CO via CORM-A1 accounted for positive alterations in the expression of miR-34a-5p and transcription factors/inhibitors (P53, NF-κB, ZEB1, SNAI1, and STAT3) and DNA methylation pattern, thereby lowering its countenance in atherogenic milieu. Inhibition of miR-34a-5p expression resulted in restoration of SIRT-1 levels and of mitochondrial biogenesis. CORM-A1 supplementation further accounted for improvement in cellular and mitochondrial antioxidant capacity and subsequent reduction in ROS. Further and most importantly, CORM-A1 restored cellular energetics by improving overall cellular respiration in HUVECs, as evidenced by restored OCR and ECAR rates, whereas a shift from non-mitochondrial to mitochondrial respiration was observed in atherogenic MDMs, evidenced by unaltered glycolytic respiration and maximizing OCR. In agreement with these results, CORM-A1 treatment also accounted for elevated ATP production in both in vivo and in vitro experimental models. Cumulatively, our studies demonstrate for the first time the mechanism of CORM-A1-mediated amelioration of pro-atherogenic manifestations through inhibition of miR-34a-5p expression in the atherogenic milieu and consequential rescue of SIRT1-mediated mitochondrial biogenesis and respiration.

## 1. Introduction

Atherosclerosis is a major cause of cardiovascular disorders (CVD) characterized by excessive lipid uptake, culminating in endothelial cell activation and foam cell formation [1,2]. The atherogenic lesion is a site of robust synergistic metabolic changes where excessive reactive oxygen species (ROS) formation and decreased antioxidant ability lead to oxidative stress. Not surprising, pathologies with altered redox homeostasis, such as hypertension, diabetes, dyslipidemia, smoking, etc., are recognized contributing factors of atherosclerosis [3].

Mitochondrial dysfunction is a key contributing factor to oxidative stress and cellular fate in atherogenesis [4]. Impaired mitochondrial redox homeostasis is determinant in the progression of early atherosclerosis in response to LDL oxidation, altered cellular respiration, and redox homeostasis [5]. Chronic and excessive mitochondrial ROS results in mtDNA damage accompanied with reduction in mtDNA-encoded subunit complexes I, III, IV, and V and impaired mitochondrial function endorsing apoptosis and release of inflammatory factors [6].

Cellular ROS exacerbates atherogenesis by regulating the expression of multiple genes and epigenetic factors [7,8]. SIRT-1, a NAD^+^-dependent deacetylase, is a redox-sensitive enzyme that acts as a robust inhibitor of oxidative stress. Induction of SIRT-1 mediates ROS reduction via a complex signaling system involving several pathways [9]. Synchronous operation of these physiological pathways culminates in the production of vital antioxidants including catalase, SOD2, and TrxR2, maintaining redox homeostasis and subsequently impending cellular inflammatory response [10,11]. SIRT-1 primarily deacetylates peroxisome proliferator-activated receptor gamma coactivator-1 alpha (PGC-1α), a central regulator of mitochondrial biogenesis and subsequent oxidation of other energy metabolic substrates [12]. Abnormal levels of ROS generated in the atherogenic milieu compromise SIRT-1 activity, thus altering mitochondrial biogenesis and function [13].

Evidence is provided that certain miRNAs can be induced by oxidative stress. miRNAs are an evolutionarily conserved class of small noncoding RNAs (22–24 nucleotides in length) that act as posttranscriptional regulators of gene expression by binding to the 3′-untranslated regions (3′-UTR) of mRNA and downregulating the target gene in diverse biological systems [14]. miR-34a-5p is a redox-sensitive miRNA of which upregulation is known to aggravate several pro-atherogenic manifestations through impending the expression of key metabolic genes and endothelial cell senescence [15,16,17,18,19]. Inhibition of miR-34a has been shown to improve dyslipidemia, atherosclerosis, and non-alcoholic fatty liver in ApoE KO or Ldlr KO mice [20]. Inferred from the existing literature, miR-34a-5p can plausibly play a crucial role in the atherogenic progression.

Vascular biology is critically regulated by endogenous gasotransmitters such as: carbon monoxide (CO), nitric oxide (NO), and hydrogen sulphide (H_2_S). In humans, CO is produced as a by-product of heme degradation. CO binds to multiple mitochondrial proteins and acts in response to cellular redox status [21,22]. Physiologically, CO is vital in mediating cell–cell interaction and maintaining vasomotor tone [23]. Perturbations in levels of CO coupled with other gasotransmitters can alter vascular physiology. CO has been reported to elevate the expression of pro-survival miRNAs in neonatal heart and in myocardial injury [24]. 

Herein, we have assessed the effects of CO-releasing molecule-A1 (CORM-A1) on atherogenic manifestations. CORM-A1 is an organometallic compound with a boron core that facilitates slow and controlled release of CO (t_1/2_ = 21 min) [25]. The therapeutic efficacy of CORM-A1 is well established in several disease models including diabetes, myocardial infarction, non-alcoholic steatohepatitis, and posterior uveitis [26,27,28]. However, the effects of CORM-A1 in alleviating atherogenic progression and miRNA-based regulation remain uninspected. In this study, we report that CORM-A1 ameliorates pro-atherogenic manifestation by miR-34a-5p mitigation and subsequent improvement in mitochondrial biogenesis and cellular redox status.

## 2. Material and Methods 

### 2.1. Chemicals and Reagents

HiEndoXL cell expansion medium (AL517), fetal bovine serum (FBS), EnVzyme, Bovine serum albumin (BSA), RPMI 1640 media, and antibiotic–antimycotic solution were purchased from Hi-media Laboratories (Mumbai, India). The iScript cDNA synthesis kit and SYBR Green Master mix were procured from Bio-Rad (Hercules, CA, USA). The miScript II RT kit and miRNeasy mini kit were purchased from Qiagen (Hilden, Germany). Antibodies SIRT-1 (SC-74465) and secondary anti-mouse (sc-516102) were procured from Santacruz biotechnology Inc. PGC-1α (ab54481), RNA-later stabilizing solution were purchased from Ambion Inc. (Naugatuck, CT, USA). β-actin (PA1-183), secondary anti-rabbit (32460), ox-LDL (L34357), and TRIzol were procured from Invitrogen (Carlsbad, CA, USA). CORM-A1, hematoxylin, eosin, Direct Red 80, and phorbol 12-myristate 13-acetate (PMA) were purchased from Sigma Aldrich (St. Louis, MO, USA). Methanol, dimethyl sulphoxide (DMSO), and 3-(4,5-dimethylthiazol-2-yl)-2,5-diphenyl tetrazolium bromide (MTT) were purchased from Sisco Research Laboratory pvt. Ltd. (Mumbai, India).

### 2.2. Animal Studies and Experimental Protocol

Male Sprague Dawley (SD) rats (6–8 weeks) were procured from Sun Pharmaceuticals Pvt. Ltd., Mumbai, India and maintained as per CPCSEA standard guidelines (23 ± 2 °C, LD 12:12, laboratory chow diet and water ad libitum), followed by a weeklong acclimatization. The experimental protocol was approved by the Animal Ethical Committee (IAEC) (Approval No. MSU-Z/IAEC030/03-2019), and experiments were conducted in the CPCSEA-approved animal house facility of the Department of Zoology, The Maharaja Sayajirao University of Baroda, Vadodara (827/GO/Re/S/04/CPCSEA). All the experiments were conducted as per ARRIVE guidelines.

Rats weighing 300 ± 20 g were randomly divided into 4 groups (*n* = 6/group). Group I (control) rats fed on a laboratory chow diet; Group II (ath) was administered a single dose of vitamin D3 (600,000 IU/Kg, i.p.) at the start of the experiment and fed on an atherogenic diet (ath diet; 3% cholesterol, 0.5% cholic acid, 0.2% 6-propyl 2-thiouracil, 5% sucrose, 10% lard, and 81.3% powdered laboratory chow) for 6 weeks [29,30,31,32,33,34,35,36]; and Group III (ath + CORM-A1) and Group IV (ath + iCORM-A1) rats made atherogenic as mentioned above and were administered with CORM-A1/iCORM-A1 (2 mg/kg; i.p.) daily from the 2nd week till 6 weeks [26,37]. Rats were fasted overnight, and blood was collected the next day by retro-orbital sinus puncture (under mild isoflurane anesthesia) and centrifuged (at 4 °C and 3000 rpm for 15 min) for serum separation. Thoracic aortae were flushed with 0.9% saline, divided into three parts, and stored in RNA-later (for gene and miRNA expression study), 4% PFA (for histopathological studies), or at −80 °C (for protein studies). Enface assay was performed with intact aorta (*n* = 2) that were photographed with a Nikon D5300 camera.

### 2.3. Serum Lipid Profile

Serum lipid profile (Total Lipid (TL), Total Cholesterol (TC), Triglycerides (TG), Low-density lipoproteins (LDL), Very-low-density lipoprotein (VLDL), and cholesterol/high-density lipoprotein (CHL/HDL), LDL/HDL ratio) were estimated using commercially available kits (Reckon Diagnostic kits, Vadodara, Gujarat, India). The atherogenic index of plasma (AIP) was calculated using the formula log_10_ (TG/HDL) and cardiac risk ratio (CRR) was calculated as TC/HDL.

### 2.4. Histopathological Study

Samples of thoracic aorta (*n* = 6/group) were dehydrated and embedded in paraffin wax blocks, and serial sections of 5 μm were cut using a microtome. Sections were stained with hematoxylin and eosin (H&E) or Picrosirius red to observe gross histopathological changes or collagen derangement, respectively, in thoracic aorta. The same were photographed on a Nikon eclipse Ti2-E (Tokyo, Japan) microscope. Elastin autofluorescence was observed (for derangement and fragmentation) and photographed using a FLoid imaging station (Thermo Fisher Scientific, Waltham, MA, USA). Collagen and elastin contents were quantified using FiJi software (ImageJ, NIH, Bethesda, Rockville, MD, USA), and investigators blinded to this study determined the intima–media thickness (IMT). Also, aortic stiffness was calculated as the collagen/elastin ratio. 

### 2.5. Immunohistochemical Analysis 

Note that 5 µm-thick sections of thoracic aortas were subjected to immunohistochemical (IHC) staining for detection of SIRT-1, ICAM-1, HSP60, and CD68^+^. Briefly, paraffin-embedded sections were de-paraffinized in xylene, and re-hydrated in graded series of ethyl alcohol. After antigen retrieval, sections were blocked in 1% fetal bovine serum (FBS) for 30 min at RT and incubated overnight with primary antibodies (SIRT-1, HSP60, and ICAM-1 at 1:200; and CD68^+^ at 1:100) at 4 °C in a humidified chamber. Later, the sections were incubated with horseradish peroxidase (HRP) conjugated secondary antibody (Dako, Agilent, Santa Clara, CA, USA) for 1 h at RT. Further, DAB substrate (Dako, Agilent, Santa Clara, CA, USA) was added followed by counter-staining with hematoxylin. Sections were observed, and images were captured on a Nikon eclipse Ti2-E (Tokyo, Japan) and positively stained regions were quantified using Fiji software (ImageJ, NIH, Bethesda, MD, USA).

### 2.6. Cell Culture and Treatment 

HUVEC cells (HiMedia Laboratories, Mumbai, India) were cultured in HiEndoXL cell expansion medium supplemented with HinEndoXL Endothelial cell growth supplement and 1X antibiotic–antimycotic solution. Passaging was done at 70–80% confluency using EnVzyme. Logarithmically growing HUVEC cells (passages 2–7) were used for all the experimentations. THP-1 cells were procured from the National Centre for Cell Science (NCCS, Pune, India) and cultured in RPMI 1640 media supplemented with 10% FBS and 1X antibiotic–antimycotic solution. THP-1 cells were differentiated to macrophages (monocyte-derived macrophages; MDMs) by treating them with 100 nM of PMA for 24 h in the dark. Differentiated cells were further used for all the experiments. All the cells were maintained in a CO_2_ incubator at 37 °C and 5% CO_2_. 

### 2.7. Cell Viability Assay

Cells (HUVECs/MDMs) were seeded in 96-well plates and treated with CORM-A1 (10, 30, 40, 50, 100, and 200 μM) for 24 h. 3-(4,5-dimethylthiazol-2-yl)-2,5-diphenyltetrazolium bromide (MTT; 5 mg/mL) was added in an incomplete media, and cells were incubated in the dark for 4 h. The resultant formazan crystals were dissolved in DMSO (150 μL/well), and absorbance was measured at 570 nm using a Synergy HTX Multimode Microplate Reader (BioTek Instruments Inc., Winooski, VT, USA), and percentage cell viability (respect to untreated cells) was calculated. 

### 2.8. DNA Methylation Assay

Genomic DNA was isolated from HUVEC and MDMs using a GeneJet Genomic DNA (gDNA) purification kit (ThermoFisher Scientific, Waltham, MA, USA), as per the manufacturer’s protocol. To access the genomic methylation pattern, gDNA was deaminated using an EpiJet Bisulfite conversion kit (ThermoFisher Scientific, USA), according to the manufacturer’s instructions. The deaminated gDNA was further used as a template for running methylation-specific PCR (MSP Assay). CpG islands were determined in the promoter region of miR-34a, several hundred base pairs upstream of the precursor transcription start site, and 5 sets of primers were used for the methylated and unmethylated DNA regions each [38]. Real-time PCR was performed with the following conditions: 95 °C for 10 min, followed by 40 cycles of 95 °C for 30 s, 52 °C for 30 s, and 60 °C for 30 s. A reaction tube w/o a template was used as a negative control, and all the samples were run with *n* = 3 technical replicates.

### 2.9. Cellular Oxidative Stress, Mitochondrial Mass, and Mitochondrial Membrane Potential (MMP) Assessment 

HUVECs/MDMs were stimulated with 80 μg/mL oxLDL and/or 40 μM CORM-A1 for 24 h. Control and treated cells were stained with 10 μM of 2′, 7′-dichlorofluorescein (H2DCFDA; for intracellular oxidative stress)/50 nM MitoTracker with 1 μg/mL Hoechst 33,342 (for mitochondrial mass) or with JC-1 (5 μg/mL; for mitochondrial membrane potential). Stains were added to control and treated cells in fresh incomplete media for 15 min/30 min, respectively, at 37 °C, washed with 1X PBS and photographed on a FLoid Cell imaging station (Thermo Fisher Scientific, USA). 

### 2.10. Mitochondrial DNA Copy Number

Mitochondrial DNA (mtDNA) was used to determine mitochondrial density by q-PCR. Total DNA was isolated from the cells (HUVECs and MDMs) and tissue (aorta) using a GeneJET genomic DNA purification kit, according to the manufacturer’s instructions (Thermo Scientific, Waltham, MA, USA). The mtDNA copy number was calculated as the ratio of ND1 (mitochondrial encoded gene) to Nuclear18s rRNA for cells and beta-globin for aorta (nuclear-encoded gene). 

### 2.11. ATP Assay

Mitochondrial function was evaluated by a total ATP determination kit (A22066, Molecular Probes, Eugene, OR, USA), as per the manufacturer’s instructions. Briefly, every reaction was set up with 1.25 μg/mL firefly luciferase, 0.5 mM D-luciferin, and 1 mM dithiothreitol in 100 mL reaction buffer, and cell/tissue lysate was added, and luminescence was measured in a Synergy HTX Multimode Reader (Bio-Tek instruments, Inc., Winooski, VT, USA). The results were expressed as arbitrary units of luminescence compared to that measured in the control group.

### 2.12. q-PCR Analysis

Total RNA content from cells and tissue were isolated using TRIzol reagent, and cDNA was synthesized using a iScript cDNA Synthesis kit (Bio-Rad, CA, USA). mRNA levels of candidate genes were quantified with qPCR (QuantStudio-3 real time PCR, LifeTechnologies, Carlsbad, CA, USA) using a SYBR Select Master Mix. The data were normalized with the internal control (18S or GAPDH) and analyzed using the 2^−ΔΔCT^ method. The miRNeasy Kit (Qiagen, Germany) was used for total miRNA isolation, and cDNA was synthesized using a miScript II RT kit (Qiagen, Germany). The levels of miRNA were quantified with qPCR (QuantStudio-3 real-time PCR, LifeTechnologies, CA, USA) using a SYBR Select Master Mix, and data were normalized to the internal control 5S and analyzed using the 2^−ΔΔCT^ method. A reaction tube w/o a template was used as a negative control, and all the samples were run with *n* = 3 technical replicates. Primer details are listed in Supplementary Tables S1 and S2.

### 2.13. Immunoblot Analysis

Cells and tissue were homogenized in ice-cold lysis buffer with 1X protease inhibitory cocktail (Sigma Aldrich, USA). Total protein content was quantified using Bradford reagent (Bio-Rad, USA). Equal amounts of protein from each sample were loaded on 10% gel for SDS-PAGE. Protein was further transferred to PVDF membrane (Bio-Rad, USA) and blocked with 5% skimmed milk (HiMedia) following overnight incubation in primary antibody (SIRT-1, 1:500; PGC-1α, 1:700) prepared in 3% BSA. Secondary antibody (anti-rabbit, 1:1000; anti-mouse, 1:5000) treatment was done for 1 h, and blots were developed using ECL reagent. The membrane was stripped using stripping buffer and was re-probed with β-actin (1:1000).

### 2.14. Mitochondrial Bioenergetics XF Assay

Mitochondrial function was assessed using the Seahorse XFp Analyzer (Seahorse Biosciences, North Billerica, MA, USA) by monitoring changes in oxygen consumption rate (OCR) and extracellular acidification rate (ECAR) of the cells, as per the manufacturer’s protocol. Briefly, cells were seeded in Seahorse Flux Analyzer mini plates (10,000 cells/well) and incubated overnight at 37 °C. Later, cells were treated as mentioned earlier. Thereafter, the culture medium was changed to XFp base medium (Seahorse Biosciences) and placed in a non-CO_2_ incubator at 37 °C. Three OCR measurements were obtained under basal conditions and upon sequential injection of 2 μM oligomycin, 2 μM fluoro-carbonyl-cyanide phenylhydrazone (FCCP) and 0.5 μM rotenone plus 0.5 μM antimycin A. OCR values were calculated from 3 min measurement cycles. The OCR measurements were adjusted to cell numbers. Glycolysis was assessed by analyzing ECAR in cells cultured in glucose-free medium after sequential addition of 10 mM glucose, 2 μM oligomycin, and 100 mM 2-deoxyglucose. The final data were obtained using the Seahorse XFp software and calculated according to their instructions.

### 2.15. Statistical Analysis

The data were expressed as mean ± SEM and analyzed by one-way analysis of variance (ANOVA), followed by Bonferroni’s multiple comparison test using GraphPad Prism 8.0.1 (San Diego, CA, USA). * *p* < 0.05, ** *p* < 0.01, *** *p* < 0.001, and **** *p* < 0.0001 when compared to the control and ^#^ *p* < 0.05, ^##^ *p* < 0.01, ^###^ *p* < 0.001, and ^####^ *p* < 0.0001 when compared to the disease group were considered to be significant.

## 3. Results

### 3.1. CORM-A1 Ameliorates Pro-Atherogenic Manifestations in Ath Diet-Fed Rats

**Figure 1:**
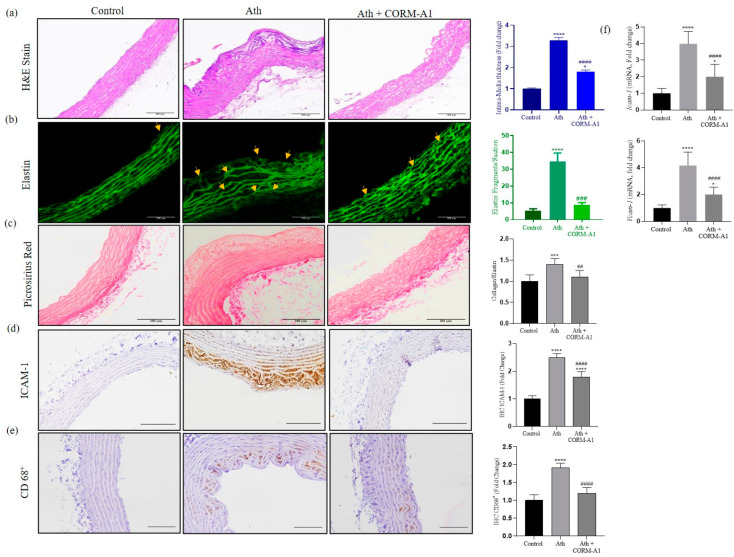
Thoracic aorta of ath diet-fed SD rats showed improvement in atherogenesis on CORM-A1 treatment. (**a**) H&E-stained section of thoracic aorta (100 µm scale) of control, ath, and ath + CORM-A1-treated SD rats and intima-media thickness (ITM) (*n* = 6/group) expressed as fold change. (**b**) Elastin autofluorescence analysis of thoracic aortae (100 µm scale); yellow arrows indicate elastin fragmentation, graphically represented as fold change. (**c**) Aortic collagen content stained with Picrosirius Red stain and arterial health assessment as collagen to elastin ratio (*n* = 6/group) represented as fold change. Immunohistochemistry of (**d**) ICAM-1 and (**e**) CD 68^+^ (100 µm scale) and their quantification represented graphically as fold change. (**f**) Transcript quantification of adhesion molecules (*ICAM-1* and *VCAM-1*) from thoracic aortae, represented as fold change (*n* = 6/group). The results are expressed as mean ± S.E.M. * *p* < 0.05, *** *p* < 0.001, or **** *p* < 0.0001 on comparison to the control and with ath diet-fed SD rat is marked with ## *p* < 0.01, ### *p* < 0.001 or #### *p* < 0.0001.

In order to closely relate to diet-induced pro-atherogenic manifestations, the experimental atherogenic model was developed by feeding an ath diet to SD rats [29,32,33]. Rats fed with the ath diet alone or in combination with CORM-A1/iCORM-A1 (i.p. injection; 2 mg/kg; [26,37]) were assessed for pro-atherogenic alterations. The kinetics of CO release from CORM-A1 and subsequent COHb formation at 2 mg/kg body weight dose is already reported and the same is considered to be nontoxic for human [25,39,40]. CORM-A1 treatment significantly improved CRP levels and serum lipid profile and congruently expressed AIP and CRR values (Figure S1b,c). Next, we assessed atherogenic manifestations in the thoracic aortae of SD rats. CORM-A1 treatment decreased arterial stiffening and lesions as evidenced in *En face* assay (Figure S1a). Further, histomorphological evaluations in H&E-stained sections showed lowered vascular derangement and intimal-media thickening in CORM-A1-treated rats (Figure 1a). Improved arterial stiffening was also observed in the CORM-A1 treatment, as demonstrated by a lowered collagen/elastin ratio, elastin fragmentation, and dampened fibrillar derangement (Figure 1b,c). The results obtained in the iCORM-A1-treated group were comparable to the ath diet-fed SD rats (data not shown), thus confirming inactivated form fail to elicit any response in the system. The presentation of adhesion molecules on activated endothelial cells facilitates monocyte recruitment to the lesion area. Herein, immunohistochemical analysis revealed that CORM-A1 treatment reduced the expression of ICAM-1 and CD68^+^ (macrophage marker; Figure 1d,e) in corroboration with lowered *ICAM-1* and *VCAM-1* mRNA transcripts (Figure 1f), demonstrating the anti-atherogenic effect of CORM-A1. These data shows that CORM-A1 treatment improved serum lipid profile, fibrillar derangement, vascular stiffening, lesions, and monocyte recruitment, thus lowering pro-atherogenic changes in ath diet-fed SD rats.

### 3.2. CORM-A1 Ameliorates Atherogenic Changes in ox-LDL Treated HUVEC and MDMs

**Figure 2:**
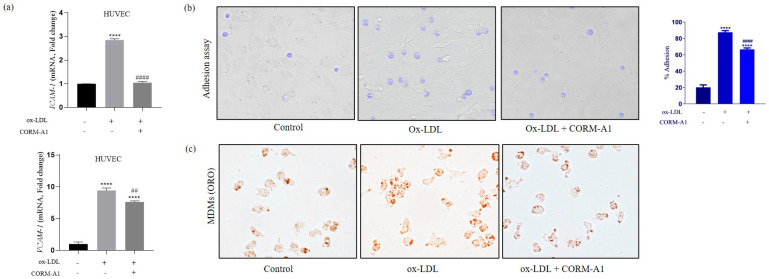
CORM-A1 treatment to ox-LDL-dosed atherogenic HUVEC and MDMs shows anti-atherogenic changes. HUVECs and Thp-1 derived MDMs were treated with ox-LDL and/or CORM-A1 for 24 h. (**a**) Transcript quantification of adhesion molecules (*ICAM-1* and *VCAM-1*) of control, ox-LDL, and ox-LDL + CORM-A1-treated HUVECs, represented as fold change. (**b**) Microscopic images of adhesion assay performed with ox-LDL and/or CORM-A1-treated HVUEC (24 h), laid with THP-1 cells for 4 h and washed with 1X PBS. Adhered monocytes were photographed using a Floid Imaging station (20X), quantification is represented as fold change in % adhesion. (**c**) Microscopic images of Oil Red O (ORO) staining on control, ox-LDL, and ox-LDL + CORM-A1-treated MDMs (20X). The results are expressed as mean ± S.E.M. **** *p* < 0.0001, on comparison to control and to ox-LDL-treated cells is represented with ## *p* < 0.01 or #### *p* < 0.0001.

To further evaluate the anti-atherogenic potential of CORM-A1 as observed in vivo, we treated HUVEC and MDMs with ox-LDL (80 µg/mL) to induce pro-atherogenic changes. Cell viability assay with CORM-A1 showed >95% viable cells at 40 µg/mL that also reduced physiological expression of miR-34a-5p (Figure S2a,b), therefore suggesting this concentration for the in vitro experiments. The mRNA levels of cell adhesion molecules (*ICAM-1* and *VCAM-1*) were lowered in HUVEC co-treated with ox-LDL and CORM-A1, providing primary evidence on impended endothelial cell activation (Figure 2a). These data were further corroborated by cell adhesion assays, demonstrating that ox-LDL-treated HUVEC showed a higher percentage of monocyte adhesion as compared to CORM-A1 co-treated cells (Figure 2b). Similar data were obtained in MDMs cells, where treatments with ox-LDL resulted in prominent lipid uptake, and foam cell formation, as evidenced by ORO staining, and this effect was reduced by concomitant CORM-A1 treatment (Figure 2c). These observations were crucial evidence on anti-atherogenic properties of CORM-A1 in our in vitro system.

### 3.3. CORM-A1 Alleviates miR-34a-5p Expression by Altering Its Transcription Factors and Methylation Pattern

**Figure 3:**
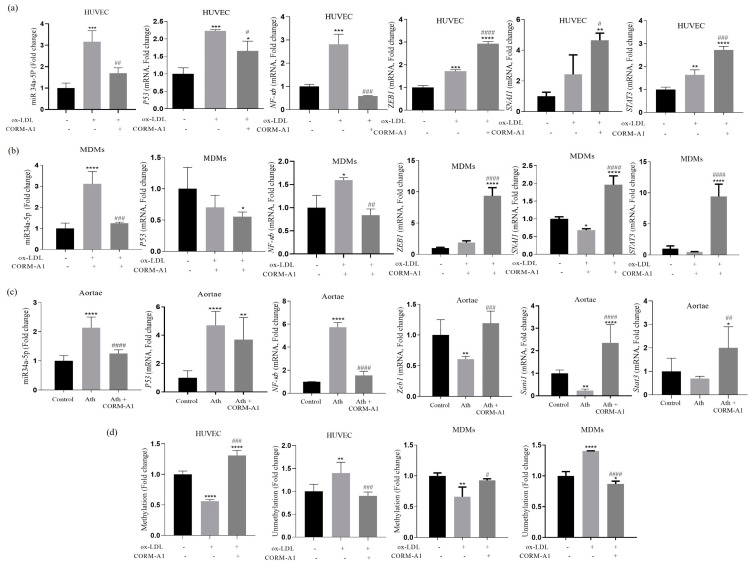
CORM-A1 lowers miR34a-5p expression in all the atherogenic experimental models by altering its transcription factors and restoring methylation in its promoter region. HUVECs and MDMs were treated with ox-LDL and/or CORM-A1 for 24 h. Transcriptomal quantification of miR-34a-5p, its transcription factors (*P53* and *NF-κb*), and transcription inhibitors (*Zeb-1*, *Snai1*, and *Stat3*) from (**a**) HUVEC, (**b**) MDMs, and (**c**) thoracic aortae SD rats from control, ath, and ath + CORM-A1 groups (*n* = 6) are represented as fold change. (**d**) Evaluation of methylation pattern in the promoter region of miR-34a-5p assessed by MSP assay in ox-LDL and/or CORM-A1-treated HUVEC and MDMs, represented as fold change. The results are expressed as mean ± S.E.M. * *p* < 0.05, ** *p* < 0.01, *** *p* < 0.001, or **** *p* < 0.0001, on comparison to the control and with atherogenic group is marked with # *p* < 0.05, ## *p* < 0.01, ### *p* < 0.001 and #### *p* < 0.0001.

High levels of miR-34a have been reported in circulation and atheromatous plaques of CVD patients [41]. A similar upregulation in miR-34a-5p was recorded in the thoracic aortae of ath diet-fed SD rats. Also, the ox-LDL-treated HUVEC and MDMs showed elevated miR-34a-5p expression, in agreement with the reports of other research groups [42,43,44]. Redox-sensitive NF-kb and P53 are transcription factors of intergenic miR-34a-5p. Herein, levels of *NF-kb* showed significant elevation in pro-atherogenic environments, that were reduced on CORM-A1 treatment. Levels of *P53* had shown elevation in the thoracic aortae of ath diet-fed SD rats and atherogenic HUVEC that were restored on CORM-A1 treatment. Transcriptional inhibitors *Zeb1*, *Snai1*, and *Stat3* showed decrement in the thoracic aortae of ath diet-fed SD rats, whereas *Zeb1* and *Stat3* titers were elevated in ox-LDL-treated HUVEC, with non-significant changes in *Snai1.* Further, atherogenic MDMs showed significant decrement in *Snai1* and *Stat3* levels. These data indicated a cell-specific response of miR-34a-5p transcriptional inhibitors to atherogenic stressors. However, CORM-A1 treatment accounted for significant elevation in expression of all the transcriptional inhibitors in thoracic aortae as well as atherogenic cells (Figure 3a–c). 

Studies have shown that CpG islands tend to undergo aberrant methylation in several pathological conditions including CVDs [45]. To determine the potential mechanism of miR-34a-5p elevation and subsequent alleviation by CORM-A1, we had analyzed the promoter region with CpG island, about 300 bp upstream of the transcription start site [38]. MSP assay of ox-LDL-treated atherogenic HUVEC and MDMs revealed hypomethylation characterized by significantly lowered methylation and elevated unmethylation. This condition is instrumental in facilitating a higher transcription rate culminating in upregulation of miR-34a-5p. The same was reverted in the CORM-A1 co-supplemented group, implying towards re-methylation of the CpG island in the promoter region that facilitates a lowered rate of transcription and miR-34a-5p expression (Figure 3d).

### 3.4. CORM-A1 Lowered miR-34a-5p Expression Correlates with Rescue of SIRT-1 Expression and Improved Mitochondrial Biogenesis

**Figure 4:**
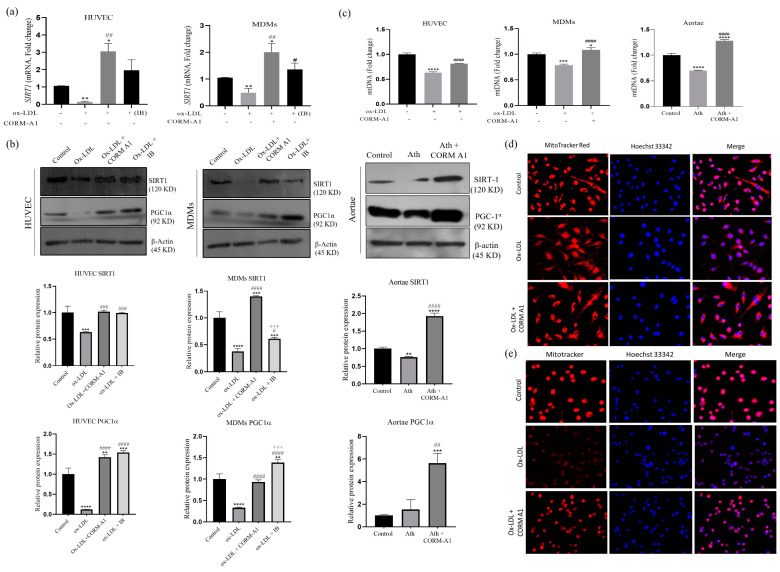
CORM-A1 raises mitochondrial mass in all the atherogenic experimental models. HUVECs and MDMs were treated with ox-LDL and/or CORM-A1 for 24 h. (**a**) mRNA expression and (**b**) Immunoblot images for SIRT1, PGC1α, and housekeeping β-actin in HUVEC and MDMs transfected with 50 nM of miR-34a-5p antagomir (IB) and thoracic aortae of SD rats (*n* = 5/group). (**c**) Mitochondrial DNA was quantified as ratio of ND1/18s rRNA for HUVEC and MDMs and ND1/beta-globin for thoracic aortae of SD rats, represented as fold change. Mitochondrial mass was assessed with MitoTracker Red staining in (**d**) HUVEC and (**e**) MDMs, co-stained with nuclear Hoechst33342 stain. The results are expressed as mean ± S.E.M. * *p* < 0.05, ** *p* < 0.01, *** *p* < 0.001, or **** *p* < 0.0001, on comparison to the control and with the atherogenic group is marked as # *p* < 0.05, ## *p* < 0.01, ### *p* < 0.001 and #### *p* < 0.0001, and with CORM-A1 treatment group is marked as +++ *p* < 0.001.

Supraphysiological levels of cellular ROS impair the expression and activity of SIRT-1 that is a central regulator of cellular homeostasis and mitochondrial biogenesis [46]. Our data showed that the atherogenic stimuli led to upregulation of miR-34a-5p, which exhibits Watson–Crick base pair complementarity to the 3′UTR of *Sirt-1* and has been shown to inhibit its expression [15,42,47]. As CORM-A1 halts miR-34a-5p expression in atherogenic conditions, we tested whether this effect was indeed associated with SIRT-1 upregulation. miR-34a-5p inhibition was achieved by transfecting cells (HUVECs and MDMs) with a specific antagomir (making IB HUVEC and IB MDMs). These cells were treated with ox-LDL and a comparison was drawn with ox-LDL-treated untransfected cells. The results showed that CORM-A1 and miR-34a-5p IB rescued SIRT-1 expression that was downregulated in atherogenic HUVEC and MDMs (Figure 4a). Immunoblot analysis further confirmed the effects of CORM-A1 and miR-34a-5p IB in rescuing SIRT-1 protein levels in both HUVECs and MDMs. As indicated before, SIRT-1 is a central regulator of mitochondrial biogenesis and function, and its restoration by CORM-A1 is likely to affect these key mechanisms at the molecular level. To confirm this, our analysis assessing protein levels of PGC-1α demonstrated an expression pattern similar to SIRT-1 in all the experimental conditions tested (Figure 4b).

**Figure 5:**
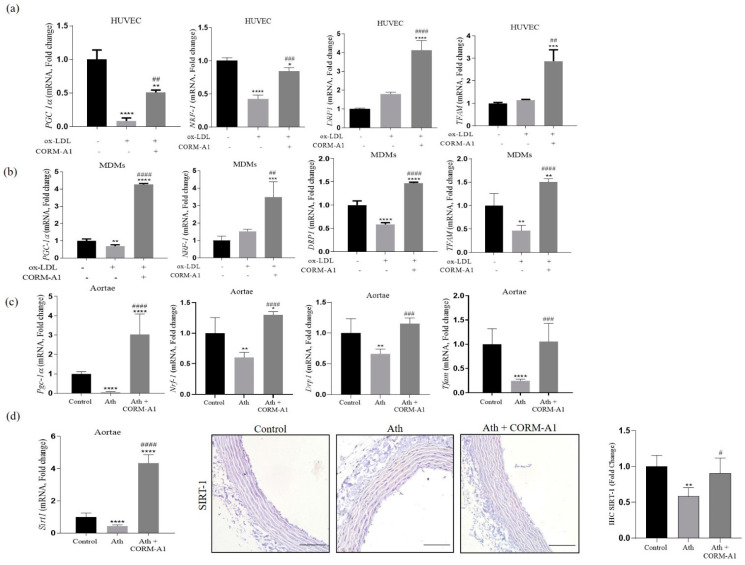
CORM-A1 elevates mitochondrial mass by elevating mitochondrial biogenesis genes in all the atherogenic experimental models. Transcriptomal quantification of mitochondrial biogenesis genes in (**a**) HUVEC, (**b**) MDMs, and (**c**) thoracic aorta of SD rats. (**d**) mRNA, Immunohistology and its quantification for SIRT1 in thoracic aortae represented as fold change (100 µm scale). The results are expressed as mean ± S.E.M. * *p* < 0.05, ** *p* < 0.01, *** *p* < 0.001, or **** *p* < 0.0001, on comparison to the control and with the atherogenic group is marked with # *p* < 0.05, ## *p* < 0.01, ### *p* < 0.001, or #### *p* < 0.0001.

Cellular mitochondrial mass was further assessed by MitoTracker Red staining. ox-LDL-treated cells exhibited lowered mitochondrial mass, that was restored in CORM-A1 co-supplemented groups (Figure 4d,e). Quantification of mtDNA copy number from the cells and thoracic aortae also showed restored mitochondrial number in response to CORM-A1 treatment (Figure 4c). To better gauge the influence of CORM-A1 on mitochondrial mass, the genes of mitochondrial biogenesis were studied at transcript and protein levels. As expected, SIRT-1 and PGC-1α levels were significantly depleted in atherogenic cells (Figure 4a,b and Figure 5a,b) and thoracic aortae of ath diet-fed SD rats (Figure 4b and Figure 5c,d), whereas *NRF1* transcripts were elevated in in vitro atherogenic systems, but the same were found to be decreased in atherogenic aortae. CORM-A1 co-supplementation elevated expression of *SIRT-1, PGC-1α,* and *NRF1* in all the disease models, suggesting increased mitochondrial biogenesis in the treated samples. CORM-A1 treatment also accounted for elevated expression of mitochondrial fission protein *Drp1* and the mitochondrial transcription factor *Tfam,* that were decreased in atherogenic milieu in a cell specific manner (Figure 5a–c). Collectively, the data showed that CORM-A1-mediated lowering of miR-34a-5p elevated SIRT-1 expression along with other mitochondrial biogenesis genes, thus resulting in enhanced cellular mitochondrial mass.

### 3.5. CORM-A1 Abrogates ox-LDL-Mediated Mitochondrial Stress and Improves Cellular Redox Status

**Figure 6:**
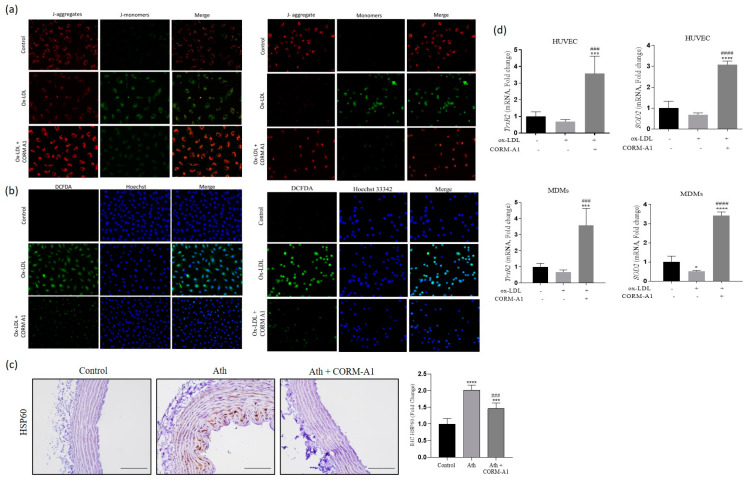
CORM-A1 improves ox-LDL-abrogated mitochondrial and subsequent cellular redox status. HUVECs and MDMs were treated with ox-LDL and/or CORM-A1 for 24 h. (**a**) JC-1 staining assessed mitochondrial membrane potential (MMP) in HUVEC (**left**) and MDMs (**right**), wherein Red fluoresced J-aggregates represents healthy mitochondrial membrane and green fluoresced J-monomers depict damaged mitochondrial membrane. (**b**) Cellular redox status was quantified by DCFDHA staining, co-stained with nuclear stain Hoechst 33,342 in HUVEC (**left**) and MDMs (**right**), wherein green fluorescence represents higher cellular ROS. (**c**) Immunohistology of HSP60 in thoracic aortae of SD rats, quantified and represented as fold change. (**d**) Transcript quantification of mitochondrial antioxidant genes (*TrxR2* and *SOD2*) in ox-LDL and/or CORM-A1-treated HUVEC and MDMs. The results are expressed as mean ± S.E.M. * *p* < 0.05, *** *p* < 0.001, or **** *p* < 0.0001 on comparison to controland with atherogenic group is marked with ### *p* < 0.001, or #### *p* < 0.0001.

Redox imbalance in vasculature culminated in elevation of miR-34a-5p, impending mitochondrial biogenesis that was ameliorated by CORM-A1 treatment. However, along with elevated numbers it was important to assess mitochondrial health for its functional anti-atherogenic implications. First, we assessed the effects of CORM-A1 by evaluating mitochondrial membrane potential (MMP). JC-1 staining showed a decrease in red/green florescence intensity ratio observed in atherogenic cells, suggestive of mitochondrial depolarization. CORM-A1 co-treated cells presented prominent red florescence emitted from J-aggregates of polarized mitochondrial membrane in healthy cells (Figure 6a). A damaged mitochondrial membrane provides a gateway to HSP 60 (DAMP) into the cytosol, resulting in endothelial dysfunction [48]. Immunolocalization study of thoracic aortae sections of ath diet-fed rats showed elevated HSP60 in the luminal and subluminal regions, that was significantly lowered in CORM-A1-treated animals (Figure 6c), implying healthy mitochondrial membrane. 

In addition, to ascertain CORM-A1-mediated mitochondrial redox response capacity, we determined the expression of mitochondria-specific antioxidant genes thioredoxin reductase 2 (*TrxR2*) and superoxide dismutase 2 (*SOD2*). Expression of *TrxR2* is known to be regulated by miR-34a-5p in endothelial cells, implying miR-34a-based regulation of mitochondrial antioxidants [19]. Herein, contemporaneous treatments of the cells with CORM-A1 significantly rescued *TrxR2* and *SOD2* mRNAs levels in atherogenic HUVEC and MDMs (Figure 6d), demonstrating improved mitochondrial redox status in cells harboring lowered miR-34a-5p. To establish whether the observed improvement in mitochondrial biogenesis and health had increased cellular detoxification capacity, we also assessed total cellular ROS status. CORM-A1 treatment significantly lowered cellular ROS as compared to ox-LDL-treated HUVECs and MDMs, as evidenced by DCFDA staining (Figure 6b). Collectively, the data are indicative of CORM-A1-mediated improvement in mitochondrial as well as cellular detoxification capacity.

### 3.6. CORM-A1-Mediated Lowering of miR-34a-5p Improves Mitochondrial Respiration and Function

**Figure 7:**
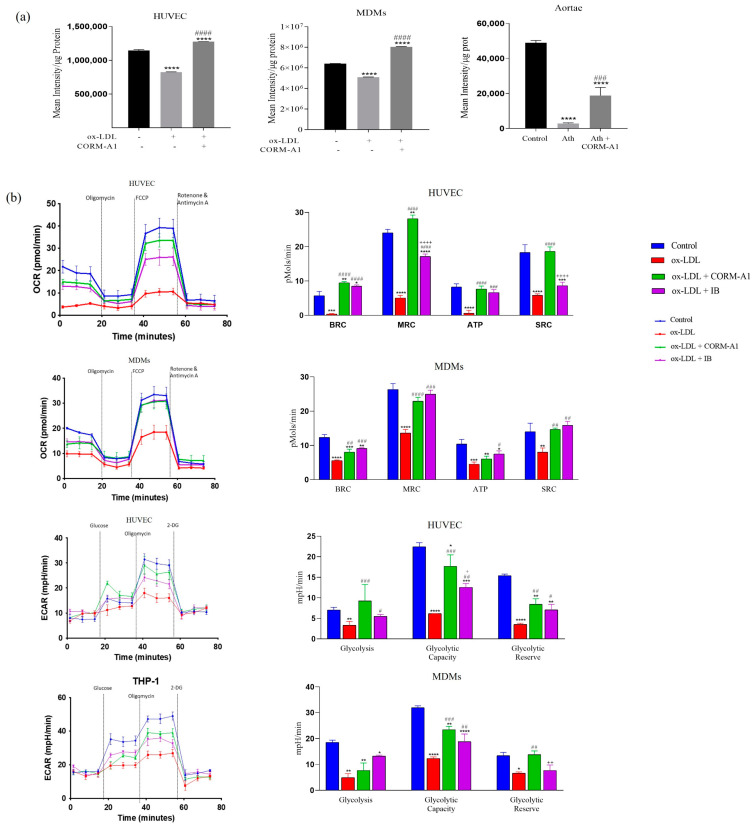
CORM-A1-mediated lowering of miR-34a-5p improves mitochondrial respiration and function. (**a**) Mitochondrial function was assessed as ATP production in HUVEC, MDMs, and thoracic aortae; ATP quantification denoted as mean intensity/µg protein. (**b**) Cells were transfected with miR-34a-5p antagomir (IB) and treated with ox-LDL and/or CORM-A1 for 24 h. Cellular respiration was measured as oxygen consumption rate (OCR) and Extracellular acidification rate (ECAR) in HUVEC and MDMs. Quantification was done using a Seahorse XFe 96 Metabolic Flux Analyzer. Quantification of other respiratory parameters from OCR is expressed as Basal Respiratory Capacity (BRC), Maximal Respiratory Capacity (MRC), ATP, and Spare respiratory capacity (SRC) and from ECAR is expressed as glycolysis, glycolytic capacity, and glycolytic reserve. The results are expressed as mean ± S.E.M. * *p* < 0.05, ** *p* < 0.01, *** *p* < 0.001, or **** *p* < 0.0001, on comparison to the control on comparison with the atherogenic group is marked with, # *p* < 0.05, ## *p* < 0.01, ### *p* < 0.001 and #### *p* < 0.0001, and with CORM-A1 treatment group is marked as + *p* < 0.05, ++ *p* < 0.01, and ++++ *p* < 0.0001.

Mitochondrial function was assessed as total ATP production. ATP content estimated in thoracic aorta of ath diet-fed SD rats and atherogenic cells exhibited decrement in total cellular ATP content. CORM-A1 treatment accounted for a significant increment in ATP levels (Figure 7a). 

miR-34a-5p is also reported to decline mitochondrial function by binding to 3′UTR of cytochrome C and lowering its expression [49,50]. Altered cytochrome C expression impairs electron transport chain functioning and compromises ATP production. Mitochondrial respiration, oxidative stress, and redox imbalance are symbolic in progression of proatherogenic changes. Herein, mitochondrial function was accessed by a Seahorse XF extracellular flux analyzer. The oxygen consumption rate (OCR) and glycolytic activity accessing lactic acid production and extracellular release (ECAR) were accessed in HUVEC and MDMs. oxLDL treatment accounted for significantly lowered OCR and ECAR values at all the time points (0–80 min). A significant improvement was observed in the oxLDL + CORM-A1 co-treated group, as evidenced by higher basal and maximal respiration capacity (BRC and MRC, respectively). Further, oligomycin mediated impact on ATP production and proton leak showed that oxLDL treatment caused a decrement in the said parameters. FCCP maximizes mitochondrial respiratory capacity and rotenone inhibits oxidative phosphorylation by blocking the complex I. This step also enables us to detect the spare respiratory capacity. The same was found to be significantly lowered in the oxLDL-treated group and improved in oxLDL + CORM-A1 co-treated cells. oxLDL treatment to miR-34a-5p antagomir transfected cells resulted in significant improvement in OCR and ECAR values, thus providing evidence on CORM-A1 orchestrating its action via inhibition of miR-34a-5p culminating in an improved mitochondrial respiration in atherogenic HUVEC and MDMs (Figure 7b).

## 4. Discussion

An overwhelming number of reports claim altered redox homeostasis as a vital etiology aiding to atherogenic progression. Key events like lipid oxidation, endothelial activation, mitochondrial dysfunction, and macrophage polarization are influenced by cellular redox status [51,52]. In this study, we report anti-atherogenic potential of CORM-A1, orchestrated by lowering expression of redox-sensitive miR-34a-5p. The study further explores the effect of CORM-A1 on mitochondrial biogenesis, respiration and its implications on adjusting cellular redox homeostasis and energetics in endothelial cells and monocytes, two cell types primarily functional in initiation and progression of atherosclerosis.

Physiologically, CO is vital in maintaining vasomotor tone, cellular homeostasis, redox status and cell–cell signaling [53]. It has colloquially received a bad connotation, because high concentrations of CO directly compete with oxygen and irreversibly bind to heme forming COHb. Uncontrolled CO exposure has been shown to manifest arrhythmia, thrombosis and other cardiovascular disorders [54]. However, delimited quantities of exogenous CO delivery have been reported to be therapeutic in various pathological conditions [55,56,57]. Previously, we reported CORM-A1-mediated activation of Nrf2/ARE pathways functional in alleviating acute and chronic liver injuries [37,58]. Other CORMs have also been reported to regulate Wnt/β-catenin, AMP kinase, and PPARγ activation to rescue endothelial cell activation [59,60]. Moreover, Qiu et al. recently reported elevated levels of endogenous CO in CVD patients [61]. Inferring from the literature, we understand that higher CO expression can plausibly be a reparatory response of a vascular system. Thus, we aim to assess anti-atherogenic potency of CORM-A1 in our experimental models.

In the present study, we demonstrate that CORM-A1 treatment improved the histoarchitecture of thoracic aortae and serum lipid profile of atherogenic SD rats. A congruent observation of miR-34a-5p elevation was made in our atherogenic models, as reported by several other groups [62,63]. Herein, we report that the CO-mediated anti-atherogenic alterations are initiated via lowering of miR-34a-5p, found to be elevated in all our atherogenic models. Being an intergenic miRNA, miR-34a-5p is autonomously transcribed by redox-sensitive NF-κb and P53 [64,65]. Exogenous administration of CORM-A1 accounted for lowered expression of these transcription factors. Whereas transcriptional inhibitors *Zeb1, Snai1* and *Stat3* were elevated unanimously in CORM-A1 treated in vitro and in vivo experimental models. In addition, aberrant DNA hypomethylation in promoter region of miR-34a-5p is reported in case of alcoholic liver injury, osteosarcoma, breast cancer, and preeclampsia [38,66,67,68]. Exploring this propensity, in our study we identified that the CpG island in the promoter region undergoes hypomethylation in atherogenic conditions facilitating miR-34a-5p transcription. CORM-A1-mediated re-methylation and lowering miR-34a-5p production observed herein is the first-time report on its epigenetic action in an atherogenic milieu. 

In the same conditions, CORM-A1-induced inhibition of miR-34a-5p expression was also paralleled by rescue of SIRT1. Direct inhibition of miR-34a-5p by transfection of the cells with its antagomir (IB) mimicked the effects of CORM-A1 in restoring SIRT-1 expression in the atherogenic milieu, further attributing CORM-A1-induced rescue of SIRT-1 to decreased miR-34a-5p level and restoration of 3′UTR activity of SIRT1. Overwhelming numbers of studies have reported SIRT1 as a molecular switch regulating endothelial health and disease by differentially controlling the expression of factors that confer anti-oxidative, anti-inflammatory, and vasodilatory functions [69]. Interestingly, Kim et al. showed that CO-mediated elevation of SIRT1 leads to inactivation of P53 and NF-kB by deacetylation to decrease inflammation and apoptosis [70]. Based on our results, we speculate that this same mechanism could explain CORM-A1 effects on transcriptional inhibition of miR-34a-5p. 

Loss of mitochondrial number has been widely associated with progression of atherosclerosis [71]. Our results show that CORM-A1 supplementation promotes SIRT1—PGC1α-mediated mitochondrial biogenesis, along with increased *NRF1, DRP1* and *TFAM* levels in both in vivo and in vitro atherogenic models. The same was evidenced by increased mitochondrial mass and mtDNA copy number. Increased cellular ROS in the atherogenic condition is reported to incur mitochondrial damage and promote further ROS production in a vicious cycle [72]. ROS induced mtDNA damage has not only been corelated to extent of atherosclerotic plaque but is also shown to precede lesion formation in young APOE KO mice [73]. We observed loss of mitochondrial membrane polarization on oxLDL treatment that was significantly restored on CORM-A1 supplementation. Sun et al. reported CORM-2 mediated alleviation of ox-LDL induced heightened mitochondrial permeability transition pore (MPTP) that resulted in leakage of cytochrome c into the cytosol [60]. On similar lines, we report inhibition in release of mitochondria homed HSP60 on CORM-A1 treatment, in the luminal and sub luminal region of thoracic aortae, accounting for its anti-atherogenic manifestation.

Impaired redox homeostasis and mitochondria also resulted in depletion of mitochondrial antioxidants *TrxR2* and *SOD2* in atherogenic conditions that were restored on CORM-A1 treatment. Studies have reported SIRT1/PGC1α mediated restoration of SOD2 and TrxR2 in endothelial cells [74,75]. Moreover, restoration of 3′UTR activity of TrxR2 gene has also been reported on miR-34a-5p inhibition [19]. The data observed in our study can plausibly be a result of combinatorial effect activated on CORM-A1 treatment.

Improved in mitochondrial copy number, higher ATP production and mitochondrial respiration implies towards efficient cellular energetics. ox-LDL accumulation in atherogenic cells, promote mitochondrial hyperpolarization and OXPHOS dysfunction [76]. In this study, ox-LDL treatment decreased BRC and MRC coupled with lowered ATP production in HUVECs and MDMs that was significantly restored on CORM-A1 treatment. Mechanistic counteractive impact of CORM-A1 was further assessed in atherogenic IB cells wherein; MDMs manifested a superior response towards the CORM-A1 mediated regulation via miR-34a-5p. HUVECs exhibit corrective variance in IB and non-IB cells, implying towards a plausible mechanism wherein; CORM-A1 mitigates via an alternative pathway [37,58,77] along with miR-34a-5p. Herein, CORM-A1 is observed to boost the overall cellular respiration in HUVECs whereas, a shift was observed in cellular energetics from non-mitochondrial to mitochondrial respiration in case of MDMs. 

## 5. Conclusions

The sum of the studies presented herein provides compelling evidence on the anti-atherogenic potency of CORM-A1 and suggests that this beneficial function involves maintenance of cellular redox homeostasis, mitochondrial biogenesis, respiration, and bioenergetics directly associated with modulation of the miR-34a-5p—SIRT-1 axis in the atherogenic milieu. Overall, the outcomes of this study support CORM-A1 and miR-34a-5p as a viable therapeutic target for atherosclerosis.

## Data Availability

All the relevant data presented in this study are available within the article and its Supplementary Materials.

## Supplementary Materials

The following supporting information can be downloaded at: https://www.mdpi.com/article/10.3390/antiox12050997/s1, Table S1: List of Rat primers, Table S2: List of human primers for qPCR, Figure S1: CORM-A1 treatment depresses atherogenic lesion and improves serum lipid profile in ath diet fed SD rats, Figure S2: CORM-A1 inhibits miR34a-5p expression with no cellular cytotoxicity.
