# Supplementary material for: CORM-A1 Alleviates Pro-Atherogenic Manifestations via miR-34a-5p Downregulation and an Improved Mitochondrial Function
Source: Antioxidants (Basel). 2023 Apr 25;12(5):997. doi: 10.3390/antiox12050997 (PMC10215967; doi:10.3390/antiox12050997)
Supplement: Supplementary file 1 [file antioxidants-12-00997-s001.zip › antioxidants-2278170-supplementary.pdf]

Table S1. List of Rat primers.

Target	Primer	Sequence (5' → 3')	Accession Number
miR34a-5p	FP	GCAGTGGCAGTGTCTTAG	MIMAT0000815
	RP	GGTCCAGTTTTTTTTTTTTTTTACAAC	
U6	FP	CTCGCTTCGGCAGCACA	XR_005498700
	RP	AACGCTTCACGAATTTGCGT	
p53	FP	ATGGGTTCCAGCACTTAGCC	XM_008767773.3
	RP	GAAGATTCCCTGGTAGCGCA	
SIRT1	FP	TATGCTCGCCTTGCTGTGGA	NM_001372090
	RP	GCTGAGTTGCTGGATTTTGTGT	
PGC-1α	FP	TTCAGGAGCTGGATGGCTTG	XM_039092489
	RP	GGGCAGCACACTCTATGTCA	
ZEB1	FP	TGCCCAAACTGCAAGAAACG	XM_039095413
	RP	GGACTGCCTGGTGATGTTGA	
SNAI1	FP	AGTTGTCTACCGACCTTGCG	NM_053805
	RP	TGCAGCTCGCTATAGTTGGG	
ICAM-1	FP	GCCTGGGGTTGGAGACTAAC	XM_039080880
	RP	CTGTCTTCCCCAATGTCGCT	
VCAM-1	FP	CCTCTCGGGAAATGCCACC	NM_012889
	RP	GTCAGAACAACGGAATCCCCA	
GAPDH	FP	CAACGGGAAACCCATCACCA	XM_039107008
	RP	ACGCCAGTAGACTCCACGACAT	
TrXR2	FP	AGGGCAGCAGAACTTTGATCT	NM_022584
	RP	GGGTTCCACATAGTCAGCCA	
SOD2	FP	CGGGGGCCATATCAATCACA	NM_017051
	RP	TAGCCTCCAGCAACTCTCCT	

Table S2. List of human primers for qPCR.

Target	Primer	Sequence (5' → 3')	Accession Number
miR-34a-5p	FP	CGAGTGGCAGTGTCTTAGCT	MIMAT0000255
	RP	CCAGTTTTTTTTTTTTTTTTTTTACAACC	
5S	FP	GGCCATACCACCCTGAACGC	6XA1_L7
	RP	CAGCACCCGGTATTCCCAGG	
18s rRNA	FP	CGTTCAGCCACCCGAGATT	7MQ9_L1
	RP	GACCCGCACTTACTGGGAATT	
SIRT1	FP	GATACCTTGGAGCAGGTTGC	NM_012238
	RP	CTCCACGAACAGCTTCACAA	
PGC1α	FP	AGTCTTCGGCTGTTTGGTGA	NM_013261
	RP	TGGAAGAACAGATGTGCCCC	
NF-κb	FP	GAGGTCTCTGGGGGTACCAT	NM_021975
	RP	AAGGCTGCCTGGATCACTTC	
P53	FP	ATTGGCCAGACTGCCTTCC	NM_001276761
	RP	TCCGGGGACAGCATCAAATC	
ZEB1	FP	GCGGCGCAATAACGTTACAAA	NM_001174096
	RP	TTCCTTTCCTGTGTCATCCTCCC	
SNAI1	FP	CTCGGACCTTCTCCCGAATG	NM_005985
	RP	TCATCAAAGTCCTGTGGGGC	
STAT3	FP	ATCACGCCTTCTACAGACTGC	NM_001384990
	RP	CATCCTGGAGATTCTCTACCACT	
SOD2	FP	GCACTAGCAGCATGTTGAGC	NM_001322815
	RP	CCGTTAGGGCTGAGGTTTGT	
TrxR2	FP	GATTAGGAGGGCGCTTCCG	NM_001352300
	RP	GTTGGGGGCATCTTGGATCA	
18s rRNA	FP	CGTTCAGCCACCCGAGATT	7MQ9_L1
	RP	GACCCGCACTTACTGGGAATT	

FP: Forward Primer; RP: Reverse Prime.

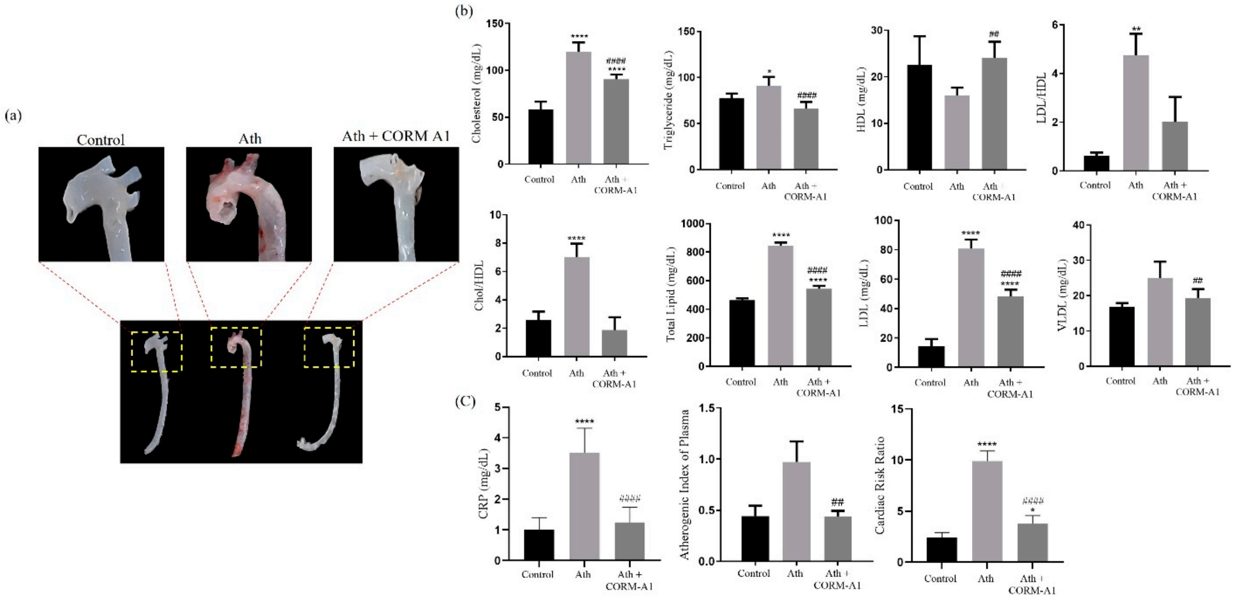

Figure S1. CORM-A1 treatment depresses atherogenic lesion and improves serum lipid profile in ath diet fed SD rats. (**a**) *En Face* Assay of thoracic aorta of SD rats comparing aorta of control, ath and ath + CORM-A1 groups. (**b**) Serum lipid profile of SD rats, represented as fold change (n=6). (**c**) Quantification of C reactive protein (CRP) and calculation of Atherogenic index of plasma (AIP) and Cardiac Risk ratio (CRR), represented as fold change. Results are expressed as mean ± S.E.M. **p* < 0.05, ***p* < 0.01, ****p* < 0.001 or *****p* < 0.0001 on comparison to control and ##*p* < 0.01, or ####*p* < 0.0001 on comparison to ath diet fed SD rats.

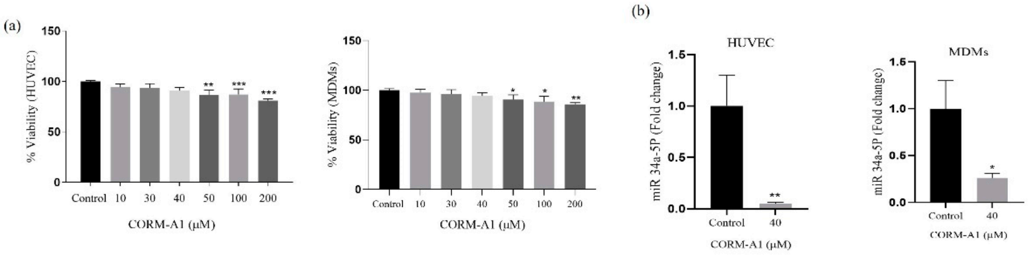

Figure S2. CORM-A1 inhibits miR34a-5p expression with no cellular cytotoxicity. (**a**) Cell viability was assessed by MTT assay using different concentrations (10, 30, 40, 50, 100 & 200 μM) of CORM-A1 for 24h in HUVEC and MDMs. (**b**) Response of physiological miR-34a-5p titers to 40 μM of CORM-A1 was assessed miRNA quantification using qPCR. Results are expressed as mean ± S.E.M. *p < 0.05, **p < 0.01, ***p < 0.001 or ****p < 0.0001 on comparison to control.
